# The impact of inclusion, dose and duration of pyrazinamide (PZA) on efficacy and safety outcomes in tuberculosis: systematic review and meta-analysis protocol

**DOI:** 10.1186/s13643-019-1231-1

**Published:** 2019-12-17

**Authors:** James D. Millard, Elizabeth A. Mackay, Laura J. Bonnett, Geraint R. Davies

**Affiliations:** 1 Wellcome Trust Liverpool Glasgow Centre for Global Health Research, Block E Royal Infirmary Complex, 70 Pembroke Place, Liverpool, UK; 2 Institute of Infection and Global Health, University of Liverpool, Liverpool, UK; 3 Africa Health Research Institute, Durban, South Africa; 4 School of Medicine, University of Liverpool, Liverpool, UK; 5 Department of Biostatistics, University of Liverpool, Liverpool, UK

**Keywords:** Tuberculosis, Clinical trials, Pyrazinamide, Efficacy, Toxicity, Safety

## Abstract

**Background:**

Pyrazinamide (PZA) is a key component of current and future regimens for tuberculosis (TB). Inclusion of PZA at higher doses and for longer durations may improve efficacy outcomes but must be balanced against the potential for worse safety outcomes.

**Methods:**

We will search for randomised and quasi-randomised clinical trials in adult participants with and without the inclusion of PZA in TB treatment regimens in the Cochrane infectious diseases group’s trials register, Cochrane central register of controlled trials (CENTRAL), MEDLINE, EMBASE, LILACS, the metaRegister of Controlled Trials (mRCT) and the World Health Organization (WHO) international clinical trials registry platform. One author will screen abstracts and remove ineligible studies (10% of which will be double-screened by a second author). Two authors will review full texts for inclusion. Safety and efficacy data will be extracted to pre-piloted forms by one author (10% of which will be double-extracted by a second author). The Cochrane risk of bias tool will be used to assess study quality. The study has three objectives: the association of (1) inclusion, (2) dose and (3) duration of PZA with efficacy and safety outcomes. Risk ratios as relative measures of effect for direct comparisons within trials (all objectives) and proportions as absolute measures of effect for indirect comparisons across trials (for objectives 2 and 3) will be calculated. If there is insufficient data for direct comparisons within trials for objective 1, indirect comparisons between trials will be performed. Measures of effect will be pooled, with corresponding 95% confidence intervals and *p* values. Meta-analysis will be performed using the generalised inverse variance method for fixed effects models (FEM) or the DerSimonian-Laird method for random effects models (REM). For indirect comparisons, meta-regression for absolute measures against dose and duration data will be performed. Heterogeneity will be quantified through the *I*^2^-statistic for direct comparisons and the *τ*^2^ statistic for indirect comparisons using meta-regression.

**Discussion:**

The current use of PZA for TB is based on over 60 years of clinical trial data, but this has never been synthesised to guide rationale use in future regimens and clinical trials.

Systematic review registration: International Prospective Register of Systematic Reviews (PROSPERO) CRD42019138735

## Background

Pyrazinamide (PZA) is a key component of modern therapy for tuberculosis (TB). It is considered an essential first-line drug and may also be used as part of treatment for multi-drug resistant disease (MDR TB). First introduced in the 1950s, its mechanism of action remains poorly understood and appears to be highly dependent on ambient pH at the site of action. PZA may be a pro-drug, undergoing conversion in vivo to pyrazinoic acid (POA) [1]. Resistance to PZA is correlated with numerous mutations in the mycobacterial *pncA* gene which codes for an amidase enzyme, PZAse, responsible for the conversion of PZA to POA [2]. Initial development of PZA suggested that it was associated with significant hepatotoxicity at the doses used [3]. Subsequent clinical trials in drug-sensitive TB (DS-TB) using a lower dose of PZA in conjunction with rifampicin demonstrated that it had an important role to play in shortening treatment, appearing to be particularly active during the first 2 months of treatment [4]. In multidrug resistant TB (MDR-TB), by contrast, PZA has been used in the absence of rifampicin for the duration of treatment and one-third to two-thirds of isolates are resistant to PZA in most series [5]. There has been renewed interest in optimising current DS-TB treatment, and a number of observational and randomised studies aim to identify the most efficacious regimens for MDR TB. Several lines of evidence suggest that increasing the PZA dose may be one route to these goals. In a murine model, increasing exposure to PZA up to fourfold (by increasing dose) resulted in improved reductions in mycobacterial load [6]. In patients, current dosing guidelines result in below ‘target’ concentrations, with low PZA exposure predicting clinical outcome [7]. At least some of the studies which demonstrated PZA’s treatment-shortening effect used milligram per kilogram (mg/kg) doses roughly double those in use today [8, 9]. Interest in optimising TB regimens by increasing the dose of PZA are tempered by concerns about hepatotoxicity and other adverse events. However, few formal dose-ranging studies of PZA have been reported, and there has been no evaluation of the risk-benefit of the drug at differing doses. Moreover, the contribution of pyrazinamide to both efficacy and hepatotoxicity may be correlated to total exposure, and hence the interaction between dose and duration may be important in making these risk-benefit analyses [10, 11]. Despite the potential importance of the question, no complete synthesis and analysis of the contribution of PZA inclusion, dose and duration to efficacy and safety outcomes in TB (of all resistance patterns) are available. We aim to perform a systematic review and meta-analysis of existing clinical trials in order to address these questions. One meta-analysis addressing the impact of PZA inclusion and dose on safety outcomes only was published almost a decade ago, but did not examine the trade-off with efficacy, identified only 29 eligible studies (our data suggest there are far more potentially eligible studies) [12] and made no adjustments for the rest of the drug regimens [13]. We hope that this work will form part of the basis for use, optimal dose and duration of PZA in clinical trials of both DS-TB and MDR-TB.

## Objectives

We aim to determine whether the following are associated with improved efficacy or worse safety outcomes during treatment of tuberculosis using first- or second-line regimens:

1. Inclusion of PZA
2. Dose of PZA
3. Duration of PZA

## Methods

This review will be conducted in accordance with the PRISMA statement [14]; a completed copy of the PRISMA-P checklist is provided in Additional file 1.

### Eligibility criteria

For efficacy outcomes, we will include randomised controlled trials and quasi-randomised controlled trials in adults with pulmonary tuberculosis (based on a microbiological diagnosis of a positive sputum smear for acid fast bacilli (AFB) or mycobacterial culture) whether fully drug-sensitive, isoniazid-resistant or MDR-TB. For safety outcomes, we considered that a microbiological diagnosis was not required, and so trials with clinical and extra-pulmonary diagnoses of TB will be included in addition. To address the objectives of this study, trials will be included if they include anti-tuberculosis regimens containing PZA and/or allow at least one of the following three comparisons:

1. For objective 1 (inclusion vs. non-inclusion of PZA): anti-tuberculosis treatment regimens which do not contain PZA (among which may be regimens considered otherwise ‘identical’: the same drugs at the same doses administered for a similar cumulative dose if given at different intervals).
2. For objective 2 (dose of PZA): anti-tuberculosis treatment regimens containing PZA at different doses.
3. For objective 3 (duration of PZA): anti-tuberculosis treatment regimens in which PZA is administered for different durations (among which PZA duration may be considered ‘identical’, if administered for a similar cumulative dose during that interval).

### Types of outcomes

Efficacy and safety outcomes will be assessed as follows:

Primary–efficacy (as per reference [12])

- Treatment failure—mycobacterial culture positive at completion of treatment protocol
- Relapse—mycobacterial culture-negative at completion of treatment protocol but culture-positive during post-treatment follow-up period

Primary–safety

- Adverse events
- Serious adverse events (as defined by the investigators)
- Discontinuation of PZA
- Regimen switch
- Deaths (all cause)
- Deaths (due to tuberculosis)

Secondary–safety

- Investigator-defined drug-induced liver injury (DILI) (whether biochemical or clinical)
- Arthralgia and joint effusions

### Information sources and search strategy

We will search for studies meeting the eligibility criteria in the Cochrane infectious diseases group’s trials register (2019), Cochrane central register of controlled trials (CENTRAL, published in *The Cochrane Library*), MEDLINE (1950 to December 2019), EMBASE (1975 to December 2019) and LILACS (1982 to December 2019). The search strategy is designed to be inclusive and identify all eligible clinical trials in tuberculosis with or without the inclusion of PZA as follows.

1. Tuberculosis AND clinical trials
2. Rifampicin OR isoniazid OR pyrazinamide OR ethambutol OR thiacetazone OR para-aminosalicylic acid OR streptomycin OR rifabutin OR rifapentine OR levofloxacin OR ofloxacin OR gatifloxacin OR moxifloxacin OR bedaquiline OR pretomanid
3. 1 AND 2

In addition, we will search the metaRegister of Controlled Trials (mRCT) and the World Health Organization (WHO) international clinical trials registry platform using ‘tuberculosis’ as the search term. We will not exclude studies based on language of publication. All studies regardless of publication status (published, unpublished, in press and in progress) will be eligible. We will hand search the reference lists of included studies and relevant reviews to identify other potentially eligible studies.

### Study selection and data extraction

A complete list of studies identified by the search strategy will be compiled and duplicates removed. One author (AM) will review the abstracts of all identified studies and remove ineligible studies, with 10% of abstracts being double-screened by a second author (JM). The full text for remaining potentially eligible studies will be accessed and reviewed by two authors (AM and JM) for inclusion. Any disagreements on eligibility will be resolved by discussion or referral to a third author (GD). Efficacy data has already been independently extracted by two authors (LB and GD) until November 2016 using a standardised electronic data extraction form. Data was collected, and inconsistent data recording was resolved through discussion and contacting the author where necessary. This search and extraction will be updated until December 2019 following the same methodology by a single author (AM), with 10% double-extracted by a second author (JM) to check for consistency. Additional safety data for all studies will be extracted to a pre-piloted supplementary electronic data extraction form for more detailed safety outcomes by a single author (AM) and 10% double-extracted by a second author (JM). We will collect data on study design, included participants, treatment regimens, outcomes and selected other data. Study authors will be contacted when the study report does not provide sufficient information concerning the study methods, treatment effect sizes or numbers of outcomes for each treatment arm. All extracted data will be entered into a spreadsheet.

### Quality assessment

Bias will be assessed using the Cochrane risk of bias tool to assess the following source of bias domains: (1) selection bias (biased allocation to interventions) via assessment of adequate sequence generation in the allocation of participants to intervention and control groups and adequate allocation concealment, (2) performance bias via assessment of blinding of personnel and participants to treatment allocation, (3) detection bias via assessment of blinding of the outcome assessor to treatment allocation, (4) reporting bias via assessment of selective outcome reporting, (5) attrition bias via assessment of the description of participants not included versus those included in the final analysis.

### Data analysis

We will calculate risk ratios as relative measures of effect for direct comparisons within trials (for all objectives) and proportions as absolute measures of effect for indirect comparisons across trials (for objectives 2 and 3 only). If there is insufficient data for direct comparisons within trials for objective 1, indirect comparisons between trials will be performed. Statistical analysis will be conducted using R for Windows, version 3.6.0. Relative and absolute measures of effect will be pooled and analysed using the package metafor. Meta-analysis will be performed using the generalised inverse variance method for fixed effects models (FEM) or the DerSimonian-Laird method for random effects models (REM) [15]. Forest plots will be generated for relative measures using metafor and absolute measures using custom code. Corresponding 95% confidence intervals (CIs) and *p* values will be calculated; results will be deemed statistically significant at *p* < 0.05. Meta-regression will be performed for absolute measures against categorised dose and duration data, including their interaction. For direct comparisons, we will quantify the level of the observed heterogeneity through the *I*^2^-statistic. For indirect comparisons using meta-regression, the *τ*^2^ statistic will be used to assess heterogeneity. We will perform the following sensitivity analyses where appropriate: (1) intention to treat (ITT, where all participants that were randomised are included), (2) per protocol (PP, where only those participants that were randomised and completed the treatment course as planned are included), (3) worst-case scenario (where participants missing an outcome are assumed to have failed or relapsed for efficacy outcomes or had a negative safety outcome) and (4) best-case scenario (where participants missing an outcome are assumed to have favourable efficacy and safety outcomes).

### Subgroup analyses

Contingent on sufficient studies, we plan to explore the following subgroups:

- HIV co-infected versus non-infected participants
- Geographical location of study (we anticipate this will be on a regional basis, dependant on the number of included studies from different locations)
- Symptomatic versus asymptomatic DILI
- DILI defined as alanine aminotransferase (ALT): aspartate aminotransferase (AST) ratio > 5 vs. ALT:AST ratio > 3

If the *I*^2^ value is over 40% and there are sufficient studies, we will explore sources of heterogeneity by stratifying on study-level characteristics including average number of drugs included in treatment regimens, use of fluoroquinolones, use of rifampicin, average number of drugs to which isolates show resistance, number of patients previously treated for TB, extent of disease and study quality.

### Assessment of publication bias

Funnel plots and Galbraith plots will be produced for all outcomes where numbers of trials (> 10) permit.

### Protocol and registration

This review has been registered with the International Prospective Register of Systematic Reviews (PROSPERO); number CRD42019138735.

## Discussion

This systematic review and meta-analysis will be the first, to our knowledge, to examine the trade-off between efficacy and safety in the use of PZA in TB, irrespective of resistance pattern. PZA is an old TB drug, but one that is likely to remain important in current and future regimens for TB and has been used in a wealth of clinical trials over the last 60 years. Correctly synthesised, this data provides the opportunity for the more rational use of PZA in future regimens and clinical trials, and hence this review has the potential for wide application. The principal practical and operational issues we foresee in this systematic review and meta-analysis of clinical trials spanning many years of publication are poor quality trials and heterogenous interventions and outcome reporting [12]. Nonetheless, this data is currently used in TB drug regimen policy-setting in a non-systematic manner, and we have planned our analysis in a rigorous manner to make the best use of this data.

## Supplementary information

**Additional file 1.** PRISMA-P checklist.

## Data Availability

All data generated or analysed during this study will be included in the published article [and its supplementary information files].

## Abbreviations

AFB: Acid fast bacilli
ALT: Alanine aminotransferase
AST: Aspartate aminotransferase
CENTRAL: Cochrane Central Register of Controlled Trials
CI: Confidence interval
DILI: Drug-induced liver injury
DS TB: Drug-sensitive tuberculosis
FEM: Fixed effects models
ITT: Intention to treat
MDR TB: Multi-drug resistant tuberculosis
mg/kg: Milligram per kilogram
mRCT: MetaRegister of Controlled Trials
POA: Pyrazinoic acid
PP: Per protocol
PZA: Pyrazinamide
REM: Random effects models
TB: Tuberculosis
WHO: World Health Organization
